# Population genetic analysis of *Giardia duodenalis*: genetic diversity and haplotype sharing between clinical and environmental sources

**DOI:** 10.1002/mbo3.424

**Published:** 2017-01-11

**Authors:** Mauricio Durigan, Maisa Ciampi‐Guillardi, Ricardo C. A. Rodrigues, Juliane A. Greinert‐Goulart, Isabel C. V. Siqueira‐Castro, Diego A. G. Leal, Sandra Yamashiro, Taís R. Bonatti, Maria I. Zucchi, Regina M. B. Franco, Anete P. de Souza

**Affiliations:** ^1^ Centro de Biologia Molecular e Engenharia Genética (CBMEG)Universidade Estadual de Campinas (UNICAMP)CampinasBrazil; ^2^ Departamento de Fitopatologia e Nematologia – ESALQ – Universidade de São PauloPiracicabaBrazil; ^3^ Departamento de Biologia AnimalInstituto de BiologiaUniversidade Estadual de Campinas (UNICAMP)CampinasBrazil; ^4^ Departamento de Patologia BásicaSetor de Ciências BiológicasUniversidade Federal do Paraná (UFPR)CuritibaBrazil; ^5^ APTA – Agência Paulista de Tecnologia dos AgronegóciosPólo Regional Centro SulPiracicabaBrazil; ^6^ Departamento de Biologia VegetalInstituto de BiologiaUniversidade Estadual de Campinas (UNICAMP)CampinasBrazil

**Keywords:** clinical samples, environmental sources, genetic diversity, *Giardia duodenalis*, haplotype sharing, population genetics

## Abstract

*Giardia duodenalis* is a flagellated intestinal protozoan responsible for infections in various hosts including humans and several wild and domestic animals. Few studies have correlated environmental contamination and clinical infections in the same region. The aim of this study was to compare groups of *Giardia duodenalis* from clinical and environmental sources through population genetic analyses to verify haplotype sharing and the degree of genetic similarity among populations from clinical and environmental sources in the metropolitan region of Campinas. The results showed high diversity of haplotypes and substantial genetic similarity between clinical and environmental groups of *G. duodenalis*. We demonstrated sharing of *Giardia* genotypes among the different populations studied. The comparison between veterinary and human sequences led us to identify new zoonotic genotypes, including human isolates from genetic assemblage C. The application of a population genetic analysis in epidemiological studies allows quantification of the degree of genetic similarity among populations of *Giardia duodenalis* from different sources of contamination. The genetic similarity of *Giardia* isolates among human, veterinary, and environmental groups reinforced the correlation between clinical and environmental isolates in this region, which is of great importance for public health.

## 1 Introduction

*Giardia duodenalis* (also known as *G. lamblia* and *G. intestinalis*) is an intestinal protozoan that parasitizes humans and many domestic and wild animals, causing giardiasis.

This disease is widespread in both developed and developing countries. It is frequently found in areas with inadequate water and sewage treatment systems and poor hygiene practices, although it has also been responsible for waterborne outbreaks in developed countries (Daly et al., 2010; Nygård et al., 2006; Robertson, Gjerde, Hansen, & Stachurska‐Hagen, 2009).

Contamination of rivers and urban streams by pathogenic protozoa in the metropolitan region of Campinas has been consistently described in several studies (Cantusio Neto, Santos, Sato, & Franco, 2010; Franco, Rocha‐Eberhardt, & Cantusio Neto, 2001; Santos, Bonatti, Cantusio Neto, & Franco, 2004). In a study that surveyed the prevalence of *Giardia* spp. in the most important regions and watersheds of São Paulo state, the highest risk of infection was estimated for the metropolitan region of Campinas (Sato et al., 2013). A recent study detected high haplotype diversity of *Giardia duodenalis* in both clinical and environmental samples in this region (Durigan, Abreu, Zucchi, Franco, & De Souza, 2014).

*Giardia duodenalis* presents significant genetic diversity, and a considerable amount of data has shown that this parasite should be considered a species complex (Caccio & Ryan, 2008). Due to this high diversity, *Giardia* isolates have been separated into eight genetic assemblages (A–H). Genetic assemblages A and B are usually associated with human infections, although these groups have also been identified in various domestic, wildlife, and marine animal species (Thompson, Hopkins, & Homan, 2000). The remaining genetic groups (C–H) are commonly found in animals and have been considered host specific, although some of these genotypes have been described in humans as well (Caccio & Sprong, 2010; Ryan & Caccio, 2013). Zoonotic transmission of *G. duodenalis* genotypes has been demonstrated experimentally, but little information exists about its occurrence in the environment under natural conditions (Plutzer, Ongerth, & Karanis, 2010). Thus, the importance of zoonotic transmissions of *G. duodenalis* needs to be elucidated (Hunter & Thompson, 2005).

Population genetics is the study of how the principles of genetics are applied to whole populations through methods that aim to quantify the variation in individuals within these populations (Hartl & Clark, 1989). Research on genetic diversity in natural populations of parasites and vectors can provide important information regarding the ecology and evolutionary potential of these organisms. For example, evidence of recombination and sexual reproduction in *Giardia duodenalis* (Cooper, Adam, Worobey, & Sterling, 2007), zoonotic transmission in *Cryptosporidium hominis* (Widmer et al., 2015), and differentiation of the community composition of crane coccidian according to the host (Honma, Suyama, Watanabe, Matsumoto, & Nakai, 2011) has been reported.

Most natural populations are subdivided into subpopulations with limited size, and the structure of a population has a substantial influence on the distribution of genetic information (de Meeûs et al., 2007). Fragmentation of parasite populations can be observed at the spatial level, the level of the host species, and the level of host individuals (Combes, 2001). The population structure of a parasite is correlated with its reproductive mode, the complexity of its life cycle, its population size, its host specificity, and the mobility of the host (Huyse, Poulin, & Théron, 2005).

In this study, genetic sequences from the protozoan *Giardia duodenalis* from clinical and environmental sources were obtained from a database to determine the genetic diversity of the parasite in a metropolitan region. The aim of this study was to use population genetics analyses to compare isolates of *G. duodenalis* from different sources and hosts to identify the degree of genetic similarity and to detect genotype sharing among these groups.

## 2 Experimental Procedures

### 2.1 Study area and design

All the DNA sequences evaluated in this study were retrieved from NCBI‐GenBank and obtained from the metropolitan region of Campinas, Sao Paulo, which comprises 20 cities and approximately 3 million people.

The sequences from clinical isolates originated from a hospital (HC), a daycare center (DC), a veterinary clinic, and the Zoonosis Surveillance Unit of the city of Campinas (VET). The sequences of environmental samples came from rivers, urban streams, sewage effluent, and hospital sewage (ENV). Table S4 presents the reference sequences obtained from NCBI that were used to confirm the genotypes of the sequences. Tables S5, S6, S7, S8, S9, S10, and S11 present all sequences used in this study and include information regarding genetic assemblages, GenBank accession numbers, and specific sites of collection.

### 2.2 Ethics statement

This study used public sequences deposited in the GenBank database. No clinical information was obtained from the isolates, and no specific permissions were required.

### 2.3 Characterization of genetic diversity through hierarchical analysis and the genealogical relationships between *Giardia duodenalis* isolates

Sequences of 530 bp from the triose phosphate isomerase gene (*tpi*), 753 bp from the beta‐giardin gene (*bg*), and 218 bp from the glutamate dehydrogenase gene (*gdh*), were obtained from GenBank. These genes have been widely used in studies on *Giardia*, which is beneficial for comparisons between different sources. These are single‐copy loci that are unlinked in the genome of *G. duodenalis* (Wielinga, Ryan, Andrew Thompson, & Monis, 2011). The lack of data from the three gene loci for most isolates determined the choice of unconcatenated analyses. The sequences were aligned using ClustalX software (Larkin et al., 2007) according to each locus. Comparisons were made between the groups and within the same genetic assemblages. Due to the wide genetic divergence between genetic assemblages, these assemblages were as considered separate populations in this study.

The genealogical relationships between sequences were estimated using TCS software (Clement, Posada, & Crandall, 2000), which unifies identical sequences into unique haplotypes and generates a network. Based on the size of the sequences present in the input file, the software calculates the maximum mutational steps for joining two haplotype groups using pairwise computations between the sequences. Because of the large number of isolates, statistical parsimony method was chosen. The groups that receive more than one haplotype present a proportional increase in size according to the number of identical sequences.

After the identification of haplotype variability in the *G. duodenalis* populations, Arlequin software (Excoffier & Lischer, 2010) which is used to evaluate the genetic diversity of populations, and the AMOVA test (molecular analysis of variance) were employed to evaluate how the genetic variability was structured in different groups of *Giardia* isolates. The genetic diversity and the degrees of similarity between groups were evaluated hierarchically by comparing clinical (sequences from the hospital, daycare, and veterinary samples) and environmental sequences.

In this study, a group of isolates was considered a set of samples from the same genetic assemblage with the same origin or collection site. Groups with less than five isolates at each locus were not considered in the genetic diversity and hierarchical analyses. Sequences of the *bg* gene were not considered in the population hierarchical analysis due to a sampling bias that prevented comparison with other groups. In the second hierarchical analysis of the distribution of genetic diversity between populations, the RIV (sequences obtained from water samples from municipal streams and rivers) and SEW (samples from sewage) groups were analyzed both separately and together with all other environmental samples (ENV Group).

## 3 Results

**Figure 1:**
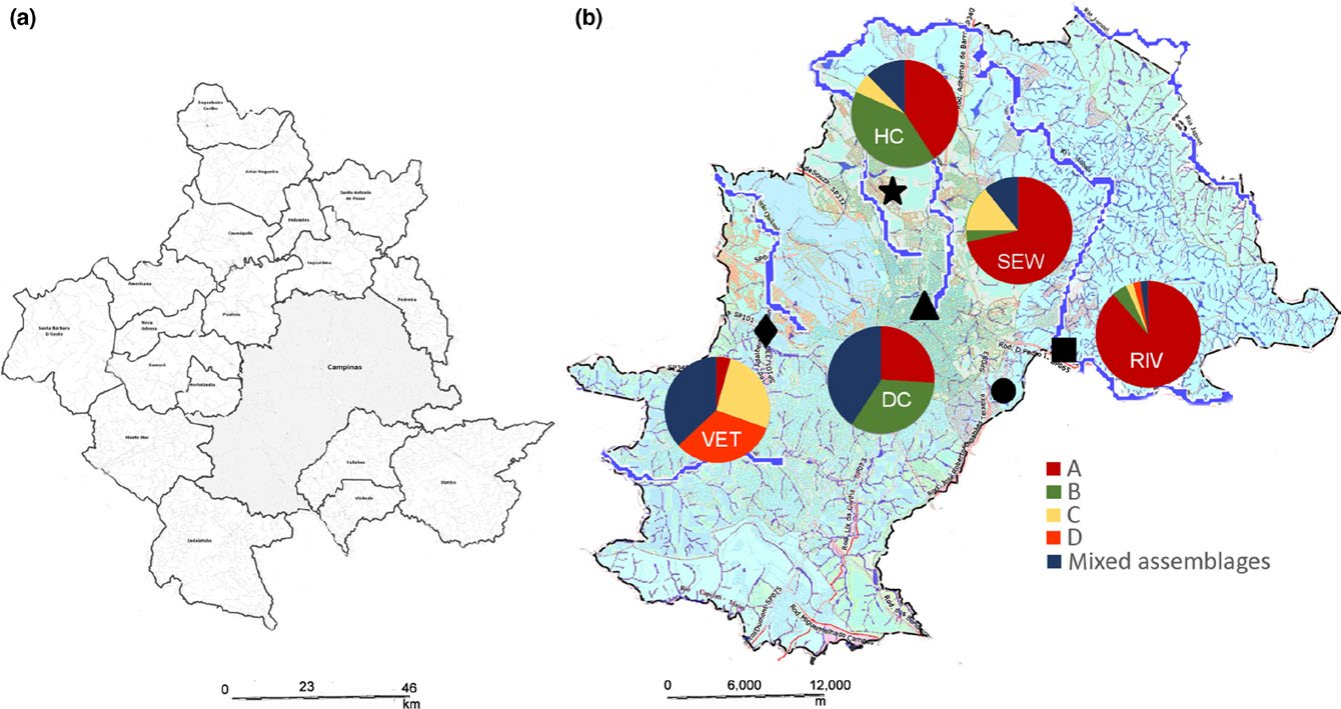
Collection sites of positive samples. Metropolitan region of Campinas (a) and the collection sites of positive samples plotted on a map of the city of Campinas (b). The genetic assemblages identified in each group were plotted in the map close to the collection site. ●, Samambaia wastewater treatment plant; ♦, Center for Zoonosis Control and Piçarrão wastewater treatment plant; ■, River Atibaia collection site;., Unicamp Hospital and hospital sewage; ▲, Daycare center, Proença and Serafim streams and river Anhumas

In total, 352 sequences from *G. duodenalis* isolates from the metropolitan region of Campinas from both clinical and environmental sources were analyzed at three different gene loci. The genetic loci *tpi*,* bg*, and *gdh* provided 175, 106, and 71 sequences, respectively. These sequences originated from 198 isolates, which were separated into the following groups: HC, for sequences obtained from a hospital; DC, for sequences obtained from a daycare center; VET, for sequences obtained from veterinary isolates; and ENV, for environmental sequences. For the analysis of haplotype diversity and genealogical relationships, the ENV group was divided into the RIV group, for sequences obtained from rivers and urban streams and the SEW group, for sequences originating from sewage (Table 1). The collection sites were plotted on a map of the city of Campinas (Figure 1). Phylogenetic analysis of each genetic locus and concatenated analysis of many isolates from different groups were performed previously as a part of an earlier study (Durigan et al., 2014).

**Table 1:** Groups of isolates of *Giardia duodenalis* from clinical and environmental sources obtained from GenBank

Group	Number of isolates	Origin
Hospital group	48	Hospital
Daycare group	28	Daycare center
Veterinary group	46	Veterinary sequences
RIV	44	Rivers and urban streams
SEW	32	Raw sewage

### 3.1 Haplotype variability

A total of 136 haplotypes were identified at the *bg*,* tpi*, and *gdh* loci in all groups. The *tpi* gene presented the greatest haplotype diversity with 75 different haplotypes, followed by the *bg* gene with 36 haplotypes. Sequences obtained from the *gdh* gene provided 25 different haplotypes. Considering the number of haplotypes according to the different sources, the HC and VET groups presented more haplotypes than the DC, RIV, and SEW groups. These data are presented in Table 2.

**Table 2:** Number of sequences and haplotypes of *Giardia duodenalis* obtained from different groups at the three loci

	*bg*	*tpi*	*gdh*
HC	24 (16)	49 (24)	37 (10)
DC	19 (5)	25 (14)	18 (12)
VET	17 (10)	66 (30)	13 (5)
RIV (ENV)	40 (6)	4 (4)	1 (1)
SEW (ENV)	6 (4)	31 (13)	2 (2)

HC, hospital group; DC, daycare group; VET, veterinary group; RIV, river group; SEW, sewage group. The number of haplotypes is presented in brackets after the number of sequences obtained per group.

### 3.2 Genealogical relationships among groups

#### 3.2.1 *tpi* gene

**Figure 2:**
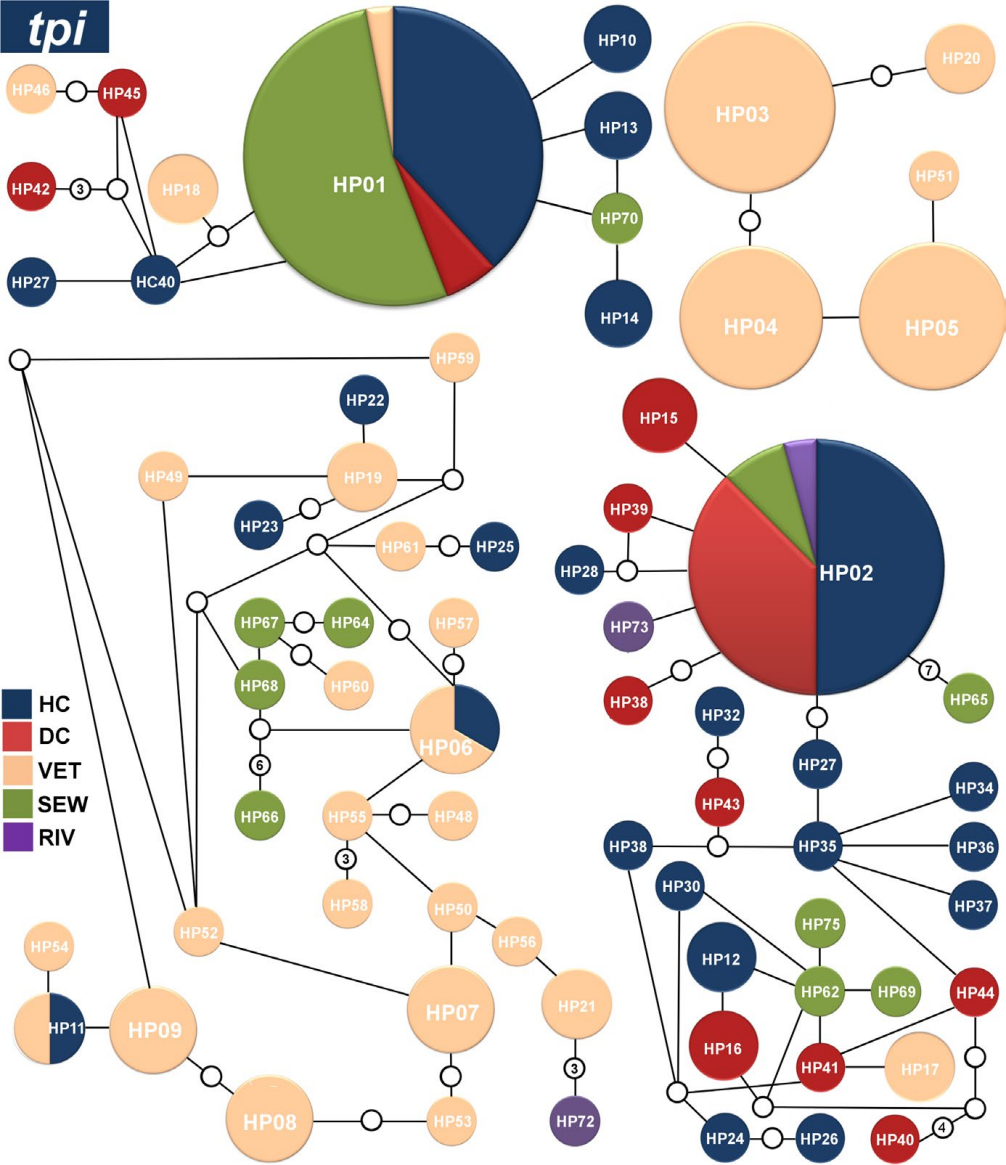
Networks of the main haplotypes of *Giardia duodenalis* at the *tpi* locus. Relative frequencies of the main haplotypes and their connections for the hospital clinical (dark blue), daycare center (red), veterinary (orange), sewage (green), and river (purple) sequences generated from a statistical parsimony network. The lines connecting each haplotype represent one mutation, and small white circles represent inferred haplotypes that were not actually observed. Haplotypes that are not connected to others exhibited more steps (or mutations) than the connection limit (95% confidence). The sizes of the circles are proportional to the haplotype frequency

The genealogical analysis of the 175 sequences of the *tpi* gene revealed the presence of 75 different haplotypes. Among these haplotypes, HP01tpi, HP02tpi, HP03tpi, HP04tpi, HP05tpi, HP06tpi, HP07tpi, HP08tpi, and HP09tpi were detected at higher frequencies at important clinical and environmental sites. These nine haplotypes together corresponded to 55% (97/175) of all sequences. Notably, the HP01tpi and HP02tpi haplotypes were detected in hospital, daycare center, and sewage sequences from the city of Campinas. The HP01tpi haplotype was also identified in a sequence obtained from a dog (Figure 2). A complete list of the haplotypes obtained for the *tpi* gene is presented in Table S1.

#### 3.2.2 *gdh* gene

**Figure 3:**
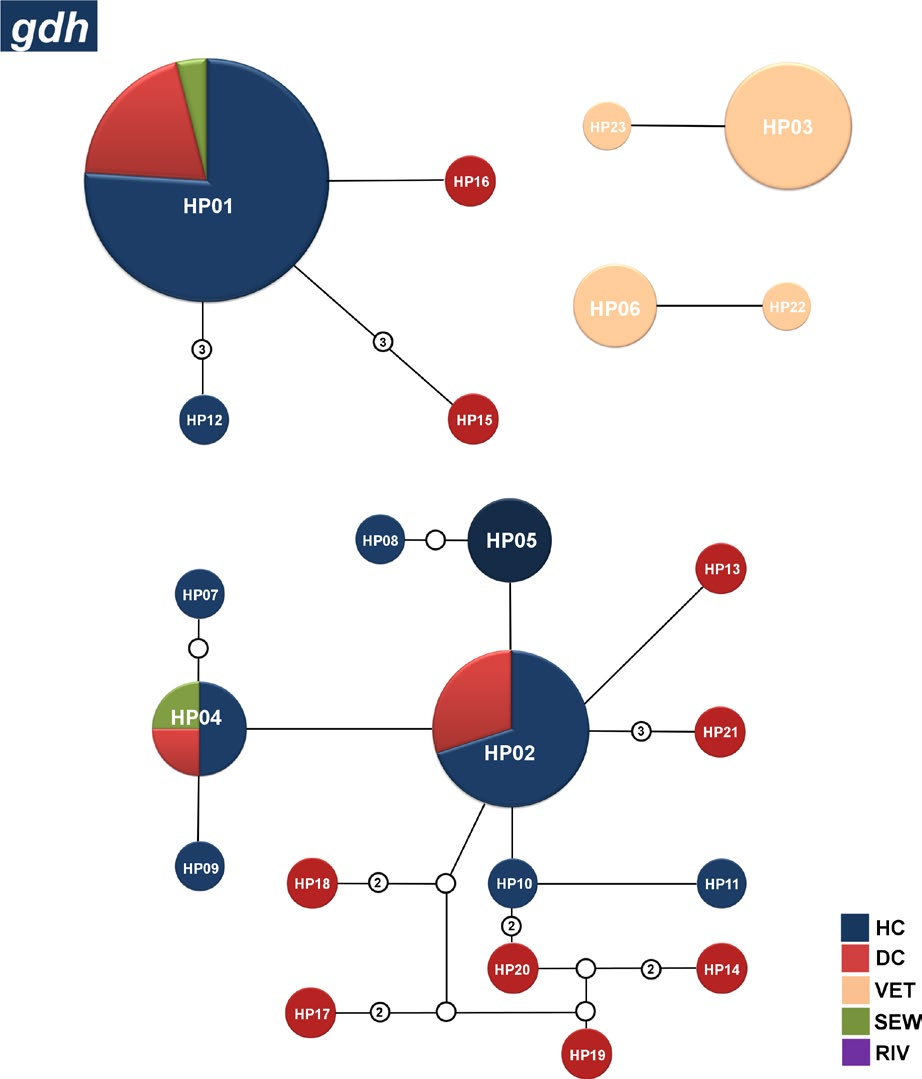
Networks of the main haplotypes of *Giardia duodenalis* at the *gdh* locus. Relative frequencies of the main haplotypes and their connections for the hospital clinical (dark blue), daycare center (red), veterinary (orange), sewage (green), and river (purple) sequences generated from a statistical parsimony network. The lines connecting each haplotype represent one mutation, and small white circles represent inferred haplotypes that were not actually observed. Haplotypes that are not connected to others exhibited more steps (or mutations) than the connection limit (95% confidence). The sizes of the circles are proportional to the haplotype frequency

The analysis of 71 sequences at the *gdh* locus revealed the presence of 25 different haplotypes among all groups. The HP01gdh, HP02gdh, HP03gdh, and HP04gdh haplotypes were identified at higher frequencies, and most of these haplotypes were also found in both clinical and environmental sites. The HP05gdh haplotype, which corresponds to genetic group C, was present in many dogs, in addition to the HP06gdh haplotype, which corresponds to genetic group D. These six haplotypes together corresponded to 73% (52/71) of all sequences (Figure 3). A complete list of the haplotypes obtained for the *gdh* gene is presented in Table S2.

#### 3.2.3 *bg* gene

**Figure 4:**
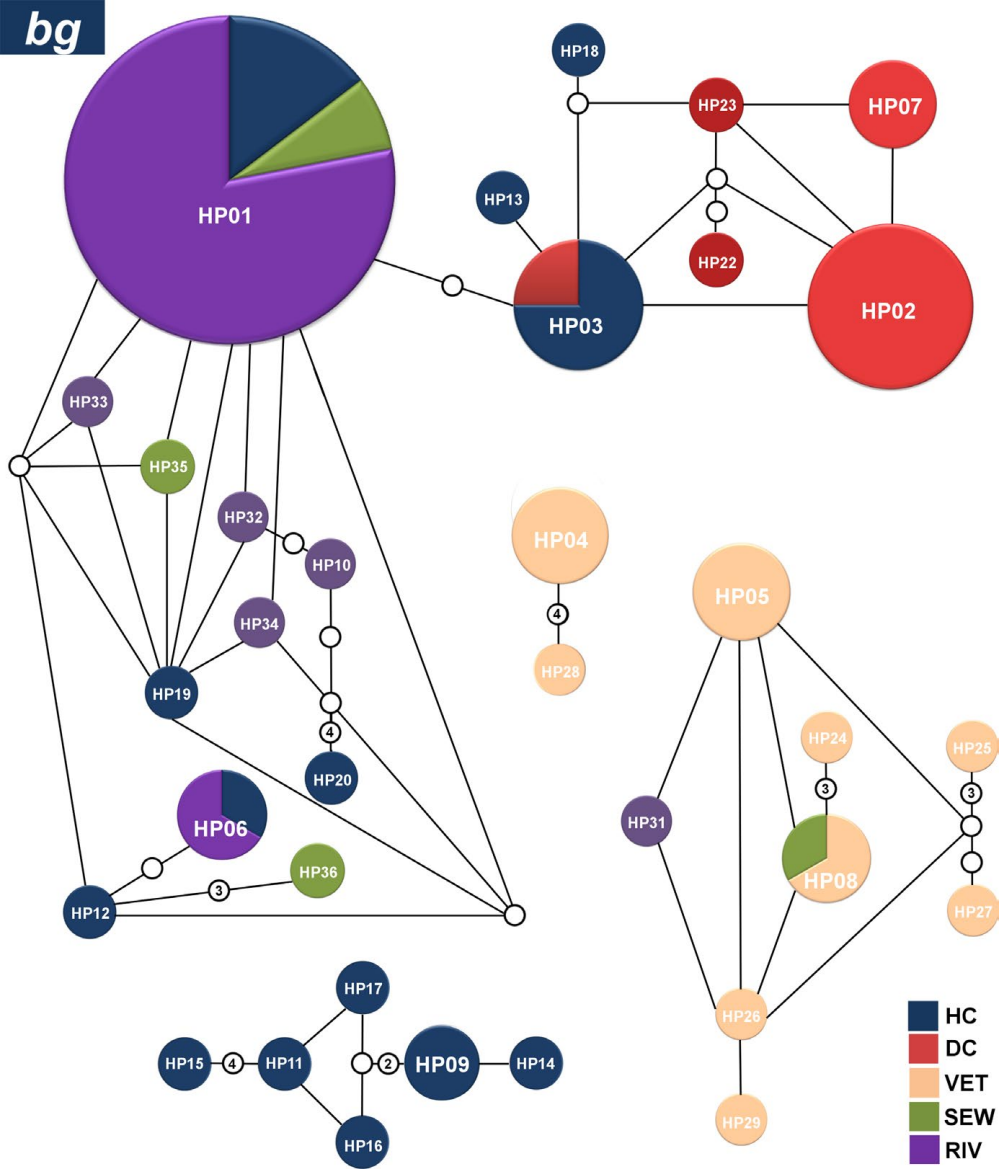
Networks of the main haplotypes of *Giardia duodenalis* at the *bg* locus. Relative frequencies of the main haplotypes and their connections for the hospital clinical (dark blue), daycare center (red), veterinary (orange), sewage (green), and river (purple) sequences generated from a statistical parsimony network. The lines connecting each haplotype represent one mutation, and small white circles represent inferred haplotypes that were not actually observed. Haplotypes that are not connected to others exhibited more steps (or mutations) than the connection limit (95% confidence). The sizes of the circles are proportional to the haplotype frequency

The analysis of 106 sequences of the *bg* gene revealed the presence of 36 different haplotypes in all groups. Haplotypes HP01bg, HP02bg, HP03bg, HP04bg, HP05bg, HP06bg, HP07bg, HP08bg, and HP09bg were detected at higher frequencies and together corresponded to 75% (79/106) of all sequences. Three haplotypes were detected at multiple sites and are highlighted as follows: the HP01 bg and HP06bg haplotypes were detected in both clinical sequences and the Atibaia River, and the HP08bg haplotype was found both in dogs and sewage sequences (Figure 4). A complete list of the haplotypes obtained for the *bg* gene is presented in Table S3.

### 3.3 Analysis of genetic diversity

The haplotype diversity of each group was also evaluated in this study. It was determined that all populations exhibited a high level of genetic diversity, which reflects the large number of haplotypes presented in Table 2. In general, all groups included individuals with considerable genetic variation, although there were exceptions in genetic group A of the RIV and SEW groups, which displayed little haplotype diversity for the *bg* and *tpi* genes (0.195 and 0.194), respectively. These results are shown in Table 3.

**Table 3:** Haplotype diversity of groups of isolates of *Giardia duodenalis*

Population	Genetic assemblage	Gene		
		*tpi*	*bg*	*gdh*
HC	A	0.636 ± 0.115	0.742 ± 0.094	0.185 ± 0.110
HC	B	0.757 ± 0.094	0.416 ± 0.190	0.816 ± 0.081
HC	C	1.000	–	–
DC	A	0.9 ± 0.161	0.105 ± 0.092	0.523 ± 0.208
DC	B	0.8 ± 0.088	–	0.945 ± 0.065
VET	C	0.960 ± 0.0213	0.4 ± 0.237	–
VET	D	0.742 ± 0.038	0.8 ± 0.113	0.25 ± 0.180
RIV	A	–	0.195 ± 0.083	–
SEW	A	0.194 ± 0.114	0.4 ± 0.237	–
SEW	B	0.9 ± 0.161	–	–
SEW	C	1.000	–	–

HC, hospital group; DC, daycare group; VET, veterinary group; RIV, river group; SEW, sewage group; –, indicates that the group did not present the minimum number of individuals to be analyzed at this locus.

### 3.4 Hierarchical analysis of the distribution of genetic diversity

The groups were compared using AMOVA to determine the variability among and within certain groups. In the established hierarchy, the initial decision was to compare the group of hospital samples (HC group) and the group of samples obtained from children at a daycare center (DC group). The descriptive data for the AMOVA analysis are provided in Table 4. The results of the comparison between the HC and DC groups showed that there were no significant variations in genetic assemblage A between these groups; thus, these groups could be considered similar.

**Table 4:** Hierarchical analysis of molecular variance (AMOVA) among human clinical sources of *Giardia duodenalis* belonging to genetic assemblages A and B

	Gene	*df*	Sum of squares	Components of variance	Percentage of variation	*p*‐value	Fixation index	Significant FST
Genetic assemblage A HC × DC								
Among populations	*tpi*	1	0.93	0.045	7.53			
Within populations	*tpi*	23	12.95	0.563	92.47			
Total		24	13.88	0.608	100	0.239	0.075	–
Genetic assemblage A HC × DC								
Among populations	*gdh*	1	0.774	0	0			
Within populations	*gdh*	26	22.333	0.858	100			
Total		27	23.107	0.858	100	0.332	0	–
Genetic assemblage B HC × DC								
Among populations	*tpi*	1	1.504	0	0			
Within populations	*tpi*	42	71.542	1.703	100			
Total		43	73.046	1.703	100	0.419	0	–
Genetic assemblage B HC × DC								
Among populations	*gdh*	1	4.751	0.253	15.75			
Within populations	*gdh*	26	35.321	1.358	84.25			
Total		27	40.072	1.612	100	0.0005	0.157	+

HC, hospital group; DC, daycare group; –, indicates that these groups are genetically similar according to the AMOVA test; +, indicates that these groups are genetically different according to the AMOVA test.

Regarding the groups that originated from genetic assemblage B, contrasting results were found between the HC and DC groups at the two markers used. For the *tpi* locus, there was no significant variation between the groups, but for the *gdh* locus, the variation between the HC and DC groups (although remarkably concentrated at the intrapopulation level) showed significant differences.

Isolates from the HC group, belonging to genetic assemblage C, were compared with isolates from the veterinary group of the same assemblage at the *tpi* locus. The results of this comparison showed that there were no significant variations between these groups; thus, these groups could be considered similar (Table 5).

**Table 5:** Hierarchical analysis of molecular variance (AMOVA) between human and veterinary sources of *Giardia duodenalis* belonging to genetic assemblage C

	Gene	*df*	Sum of squares	Components of variance	Percentage of variation	*p*‐value	Fixation index	Significant FST
Genetic assemblage C HC × VET								
Among populations	*tpi*	1	3.260	0.174	8.9			
Within populations	*tpi*	30	53.615	1.787	91.1			
Total		31	56.875	1.961	100	0.077	0.088	–

HC, hospital group; VET, veterinary group; –, indicates that these groups are genetically similar according to the AMOVA test; +, indicates that these groups are genetically different according to the AMOVA test.

At the second hierarchical level, the *G. duodenalis* groups obtained from human clinical isolates (HC and DC) were compared with those obtained from environmental isolates (ENV). The results for both genetic assemblages (A and B) indicated that these groups (HC and DC × ENV) were very similar, with no significant genetic variation being observed between them for both genetic assemblages. These results are shown in Table 6.

**Table 6:** Hierarchical analysis of molecular variance (AMOVA) between human and environmental sources of *Giardia duodenalis*

	Gene	*df*	Sum of squares	Components of variance	Percentage of variation	*p*‐value	Fixation index	Significant FST
Genetic assemblage A HC + DC) × ENV								
Among populations	*tpi*	1	1.013	0.006	0.72			
Within populations	*tpi*	43	37.52	0.872	99.28			
Total		44	38.533	0.878	100	0.256	0.007	–
Genetic assemblage B (HC + DC) × ENV								
Among populations	*tpi*	1	3.101	0.076	3.73			
Within populations	*tpi*	51	100.182	1.964	96.27			
Total		52	103.283	2.040	100	0.156	0.037	–

HC, hospital group; DC, daycare group; VET, veterinary group; –, indicates that these groups are genetically similar according to the AMOVA test; +, indicates that these groups are genetically different according to the AMOVA test.

## 4 Discussion

The structure of a population has a great influence on the distribution of genetic information (de Meeûs et al., 2007). Identifying intraspecific variation has been central to understanding the transmission dynamics of *Giardia* spp. (Thompson & Ash, 2016). In this study, the relationships between isolates of *G. duodenalis* from different sources and hosts were verified through analyses of genealogical relationships and population genetics. Our results suggested high haplotype diversity and a direct relationship between clinical and environmental populations of *Giardia* in the metropolitan region.

Knowledge concerning the genetic diversity of *G. duodenalis* is an essential component in increasing our understanding about the taxonomy, epidemiology, and population dynamics of a parasite (Huey et al., 2013). *Giardia*, as a member of the diplomonads, has always been considered asexual. However, recent studies identified core meiotic recombination machinery and variation in the frequency of the allelic sequence heterozygosity between different genomes and probable recombinant genotypes, suggesting that this protozoan may be capable of meiosis, and thus, sexual reproduction (Franzén et al., 2009; Jerlström‐Hultqvist et al., 2010; Ramesh, Malik, Logsdon, & City, 2005). Although evidence from genomic and population genetic studies suggests that meiosis may occur in Giardia, sexual reproduction has never been directly observed (Cooper et al., 2007). Due to the considerable genetic variation among the different genetic assemblages of *G. duodenalis*, recent studies have suggested major changes in the nomenclature and taxonomy of *Giardia*, assuming that these genetic assemblages likely correspond to different species (Jerlström‐Hultqvist et al., 2010; Monis, Caccio, & Thompson, 2009; Thompson & Ash, 2016). Considering the high genetic diversity of the parasite and the goal of avoiding misinterpretations or sampling bias each genetic assemblage was considered a distinct group in our study and population comparisons were performed between isolates that belonged to the same genetic assemblage.

One limitation of population genetic studies is the complexity of comparing populations of microorganisms. The lack of availability of data for the main molecular markers within particular isolates and the absence of certain genetic assemblages in some populations can prevent further comparisons between groups of *G. duodenalis*. In this study, some populations had available data from only one (RIV) or two (SEW) molecular markers, which narrowed the possibility of comparisons with other groups. This finding may be a consequence of the low success rates of the amplification and sequencing reactions in these groups at this locus. Numerous factors, such as the DNA extraction methods or PCR inhibitors used and whether single‐copy or multi‐copy genes are targeted, influence the successful amplification of DNA (Elwin, Fairclough, Hadfield, & Chalmers, 2013).

Regarding the number of haplotypes at the *tpi*,* bg*, and *gdh* loci, as well as the number of sequences that were initially evaluated, the *tpi* gene was concluded to exhibit the greatest diversity of haplotypes, probably due to the increased number of sequences available. The smaller number of haplotypes at the *gdh* locus probably reflects the low number of sequences obtained for this marker compared with the others, as indicated in a previous study (Durigan et al., 2014) that used sequences that partially correspond to those employed in this study.

The application of molecular tools has confirmed that *Giardia* is zoonotic even though the epidemiology of zoonotic infections remains controversial (Fletcher, Stark, Harkness, & Ellis, 2012); Thompson and Ash (2016) stated that more isolates of *Giardia* from the same hosts in different geographical areas need to be characterized and the most valuable approach is to study transmission at a local level where the frequency of these protozoans is high. Our study addressed these concerns and analyzed data from multiple hosts and sources in a region of Brazil with high endemism. The genealogical relationships among groups indicated that although there is great diversity of *G. duodenalis* haplotypes, only a few of these haplotypes are overrepresented in the studied populations, which correspond to most of the sequences obtained in this metropolitan region. The networks presented in this study showed that these haplotypes are present in many populations, which suggests that some isolates may play a more important role regarding zoonotic or anthroponotic transmission than other isolates. The identification of a particular haplotype with a higher frequency in different populations is of great importance for public health.

The high level of haplotype diversity identified in this study was determined through analysis of the diversity of haplotypes and their proportion in a population. This result, although initially unexpected for a parasite that reproduces clonally, probably reflects the dynamism of the metropolitan region, which comprises more than 3 million inhabitants as well as metropolitan hospitals, some of the most reputable universities of the country, and an international airport, all of which may influence how widespread several genotypes are in this region. The river group (RIV) presented low haplotype diversity (even though its result was obtained from only one locus, *bg*), which compromises comparisons with the other groups.

Genetic similarity between the hospital and daycare center (HC × DC) groups was observed for both assemblages (A and B), which demonstrates a robust correlation between the isolates of this parasite and both hosts. Children, and particularly those who attend daycare centers, primary schools, and orphanages, are highly susceptible to infection by *G. duodenalis* (Feng & Xiao, 2011; Franco & Cordeiro, 1996; Puebla et al., 2014). The high diversity of the haplotypes observed in the daycare center sequences most likely reflects the multiple sources of contamination observed at that location, which may be associated with the socioeconomic vulnerability of a portion of the children attending these facilities (Durigan et al., 2014). The high diversity of haplotypes found in hospital samples can be explained by the fact that patients who come from different regions of the city and even from different cities, have greater chances of carrying a wide range of *Giardia* genotypes that are not related to single source of contamination, which could narrow the genetic diversity of the haplotypes detected. Our findings suggest that these groups (HC and DC) may be susceptible to the same contamination events in this metropolitan region.

The existence of five sequences belonging to genetic assemblage C in the HC group allowed us to compare these sequences with those of the same genetic assemblage from the VET group through the analysis of genealogical relationships and AMOVA. Our first result indicated genetic similarity between the HC and VET groups, which demonstrates a correlation between their isolates. The comparison between genotypes showed that two haplotypes are shared between these groups. Although genetic assemblage C is not commonly found in humans, this study found identical haplotypes of *G. duodenalis* from genetic assemblage C that are shared between humans and dogs. This result, which has also been reported in previous studies (Durigan et al., 2014; Liu et al., 2014; Soliman, Fuentes, & Rubio, 2011), is considered rare but should not be ignored because it indicates new genotypes with zoonotic potential (Sprong, Der Van, & Caccio, 2009).

Many studies have identified populations of dogs infected with zoonotic genotypes of *Giardia*, which represent potential sources of zoonotic infection in humans (Covacin, Aucoin, Elliot, & Thompson, 2011; Pallant, Barutzki, Schaper, & Thompson, 2015). Although, in our study, the group of veterinary samples differed substantially from the other groups due to the widespread presence of host‐specific genotypes, some genotypes of genetic assemblages A and B were also identified in this group. This fact allowed us to identify haplotype sharing between the veterinary, human, and environmental groups. These results led us to conclude that the veterinary group may act as a reservoir of isolates of *G. duodenalis* with zoonotic potential both for the well‐known genetic assemblages A–B and genetic assemblage C.

The comparison between human and environmental sequences (HC/DC × ENV) also showed high genetic similarity for both comparisons (genetic assemblages A and B). Many environmental sequences presented genotypes that are commonly detected in humans. Although *Giardia* contamination in this region has been widely described, only recent studies have assigned this contamination to *Giardia* genotypes that parasitize humans (Durigan et al., 2014; Santos et al., 2013). The identification of identical haplotypes between clinical and environmental sequences suggests that there should be a relationship between environmental contamination by *G. duodenalis* and clinical infection in humans. Future molecular surveillance studies may help elucidate the origin of the genetic diversity of *G. duodenalis* populations in the metropolitan region of Campinas.

## 5 Conclusions

The application of a population genetic analysis in epidemiological studies opens up new possibilities allowing quantification of the degrees of genetic similarity between populations of *Giardia duodenalis* from different sources of contamination. Our results showed that in the metropolitan region of Campinas, there is high diversity of haplotypes and substantial genetic similarity between clinical and environmental groups of *G. duodenalis*, which could be quantitatively demonstrated. The sharing of *Giardia* genotypes between human, veterinary, and environmental groups reinforced the correlation between clinical and environmental isolates in this region and allowed us to identify new zoonotic genotypes, which is of great importance for public health.

## Conflict of Interest

The authors have no conflicts of interest.
