# Supplementary material for: Population genetic analysis of Giardia duodenalis: genetic diversity and haplotype sharing between clinical and environmental sources
Source: Microbiologyopen. 2017 Jan 11;6(2):e00424. doi: 10.1002/mbo3.424 (PMC5387310; doi:10.1002/mbo3.424)
Supplement: Supplementary file 1 [file MBO3-6-na-s001.docx]

**Table S1.** Correspondence between haplotypes and sequences obtained from isolates from the *tpi* gene*.*

| **Haplotype** | | **Molecular Marker** | | **Genetic Assemblage** | | **Isolates** |
| --- | --- | --- | --- | --- | --- | --- |
| HP01 | *tpi* | | AII | | HC06 HC10 HC11 HC21 HC22 HC23 HC29 HC31 HC35 HC36 HC42 HC49 HC51 DC15 DC25 VET04 04JTPI 07JTPI 12JTPI 13JTPI 14JTPI 15JTPI 17JTPI 18JTPI 19JTPI 21JTPI 23JTPI 25JTPI 26JTPI 27JTPI 30JTPI 31JTPI 35JTPI 37JTPI | |
| HP02 | *tpi* | | BIV | | HC09 HC14 HC15 HC16 HC20 HC32 HC34 HC37 HC41 HC45 HC46 HC47 DC03 DC05 DC14 DC16 DC17 DC19 DC21 DC22 DC24 28JTPI 29JTPI ENV05TPI | |
| HP03 | *tpi* | | D | | D03RLTPI D08RLTPI D21RLTPI D22RLTPI D27RLTPI D28RLTPI D29RLTPI D30RLTPI D32RLTPI D35RLTPI D40RLTPI | |
| HP04 | *tpi* | | D | | D02RLTPI D06RLTPI D07RLTPI D13RLTPI D18RLTPI D31RLTPI D38RLTPI D39RLTPI | |
| HP05 | *tpi* | | D | | VET01L VET05L D04RLTPI D05RLTPI D09RLTPI D15RLTPI D23RLTPI D26RLTPI | |
| HP06 | *tpi* | | C | | HC19 D11RSTPI D29RSTPI | |
| HP07 | *tpi* | | C | | D01RLTPI D11RLTPI D16RLTPI | |
| HP08 | *tpi* | | C | | D04RSTPI D06RSTPI D07RSTPI | |
| HP09 | *tpi* | | C | | D17RSTPI D34RSTPI D35RSTPI | |
| HP10 | *tpi* | | AII | | HC01 HC12 | |
| HP11 | *tpi* | | C | | HC13 D27RSTPI | |
| HP12 | *tpi* | | BIII | | HC33 DC18 | |
| HP13 | *tpi* | | AII | | HC44 DC27 | |
| HP14 | *tpi* | | AII | | HC48 HC50 | |
| HP15 | *tpi* | | BIV | | DC01 DC08 | |
| HP16 | *tpi* | | BIII | | DC04 DC10 | |
| HP17 | *tpi* | | BIV | | VET01 VET05 | |
| HP18 | *tpi* | | AI | | VET02LA DC03RSTPI | |
| HP19 | *tpi* | | C | | VET02LC D41RSTPI | |
| HP20 | *tpi* | | D | | D10RLTPI D12RLTPI | |
| HP21 | *tpi* | | C | | D08RSTPI D09RSTPI | |
| HP22 | *tpi* | | C | | HC02 | |
| HP23 | *tpi* | | C | | HC04 | |
| HP24 | *tpi* | | BIV | | HC07 | |
| HP25 | *tpi* | | C | | HC08 | |
| HP26 | *tpi* | | BIV | | HC17 | |
| HP27 | *tpi* | | BIV | | HC24 | |
| HP28 | *tpi* | | BIV | | HC25 | |
| HP29 | *tpi* | | AII | | HC27 | |
| HP30 | *tpi* | | BIV | | HC30 | |
| HP31 | *tpi* | | BIV | | HC38 | |
| HP32 | *tpi* | | BIV | | HC39 | |
| HP33 | *tpi* | | AII | | HC40 | |
| HP34 | *tpi* | | BIV | | HC43 | |
| HP35 | *tpi* | | BIV | | HC43A | |
| HP36 | *tpi* | | BIV | | HC43B | |
| HP37 | *tpi* | | BIV | | HC43C | |
| HP38 | *tpi* | | BIV | | DC02 | |
| HP39 | *tpi* | | BIV | | DC06 | |
| HP40 | *tpi* | | BIII | | DC09 | |
| HP41 | *tpi* | | BIII | | DC11 | |
| HP42 | *tpi* | | AII | | DC12 | |
| HP43 | *tpi* | | BIV | | DC20 | |
| HP44 | *tpi* | | BIII | | DC23 | |
| HP45 | *tpi* | | AII | | DC28 | |
| HP46 | *tpi* | | AII | | VET02 | |
| HP47 | *tpi* | | AII | | VET06 | |
| HP48 | *tpi* | | C | | VET02L | |
| HP49 | *tpi* | | C | | VET02LB | |
| HP50 | *tpi* | | C | | D19RLTPI | |
| HP51 | *tpi* | | D | | D25RLTPI | |
| HP52 | *tpi* | | C | | D01RSTPI | |
| HP53 | *tpi* | | C | | D03RSTPI | |
| HP54 | *tpi* | | C | | D05RSTPI | |
| HP55 | *tpi* | | C | | D10RSTPI | |
| HP56 | *tpi* | | C | | D16RSTPI | |
| HP57 | *tpi* | | C | | D18RSTPI | |
| HP58 | *tpi* | | C | | D19RSTPI | |
| HP59 | *tpi* | | C | | D20RSTPI | |
| HP60 | *tpi* | | C | | D21RSTPI | |
| HP61 | *tpi* | | C | | D37RSTPI | |
| HP62 | *tpi* | | B | | 01JTPI | |
| HP63 | *tpi* | | A | | 02JTPI | |
| HP64 | *tpi* | | C | | 03JTPI | |
| HP65 | *tpi* | | BIV | | 08JTPI | |
| HP66 | *tpi* | | C | | 10JTPI | |
| HP67 | *tpi* | | C | | 16JTPI | |
| HP68 | *tpi* | | C | | 20JTPI | |
| HP69 | *tpi* | | B | | 22JTPI | |
| HP70 | *tpi* | | AII | | 33JTPI | |
| HP71 | *tpi* | | C | | 36JTPI | |
| HP72 | *tpi* | | C | | ENV02TPI | |
| HP73 | *tpi* | | BIV | | ENV03TPI | |
| HP74 | *tpi* | | BIII | | ENV04TPI | |
| HP75 | *tpi* | | BIII | | ENV06TPI | |

**Table S2.** Correspondence between haplotypes and sequences obtained from isolates from the *gdh* gene*.*

| **Haplotype** | **Molecular Marker** | **Genetic Assemblage** | **Isolates** |
| --- | --- | --- | --- |
| HP01 | *gdh* | AII | HC01 HC02 HC10 HC11 HC12 HC13 HC18 HC22 HC27 HC28 HC31 HC35 HC36 HC40 HC42 HC44 HC48 HC49 HC51 DC07 DC15 DC25 DC27 DC28 ENV01 |
| HP02 | *gdh* | BIV | HC15 HC16 HC23 HC25 HC32 HC38 HC46 DC03 DC04 DC05 |
| HP03 | *gdh* | D | D02RGDH D04RGDH D06RGDH D07RGDH D08RGDH VET01GDH VET02GDH |
| HP04 | *gdh* | BIV | HC39 HC45 DC22 ENV06 |
| HP05 | *gdh* | BIV | HC34 HC41 HC47 |
| HP06 | *gdh* | C | D01RGDH D05RGDH VET03GDH |
| HP07 | *gdh* | B | HC07 |
| HP08 | *gdh* | BIV | HC21 |
| HP09 | *gdh* | BIV | HC30 |
| HP10 | *gdh* | B | HC33 |
| HP11 | *gdh* | B | HC43 |
| HP12 | *gdh* | A | HC50 |
| HP13 | *gdh* | BIV | DC01 |
| HP14 | *gdh* | BIV | DC09 |
| HP15 | *gdh* | A | DC12 |
| HP16 | *gdh* | A | DC13 |
| HP17 | *gdh* | BIV | DC16 |
| HP18 | *gdh* | BIV | DC17 |
| HP19 | *gdh* | BIV | DC19 |
| HP20 | *gdh* | BIV | DC20 |
| HP21 | *gdh* | BIV | DC21 |
| HP22 | *gdh* | C | D03RGDH |
| HP23 | *gdh* | D | VET05GDH |
| HP24 | *gdh* | E | VET06GDH |
| HP25 | *gdh* | D | ENV05 |

**Table S3.** Correspondence between haplotypes and sequences obtained from isolates from the *bg* gene***.***

| **Haplotype** | | **Molecular Marker** | | **Genetic Assemblage** | | **Isolates** |
| --- | --- | --- | --- | --- | --- | --- |
| HP01 | *bg* | | AII | | HC36 HC40 HC42 HC48 HC50 HC31 AT02BG AT03BG AT04BG AT06BG AT07BG AT08BG AT12BG AT13BG AT15BG AT16BG AT17BG AT19BG AT20BG AT22BG AT24BG AT25BG AT26BG AT29BG AT30BG AT31BG AT32BG AT33BG AT34BG AT35BG AT37BG AT38BG AT40BG AT41BG AT42BG AT43BG AT44BG AT45BG 15JBG 16JBG 33JBG | |
| HP02 | *bg* | | AII | | DC02 DC03 DC05 DC07 DC11 DC12 DC13 DC14 DC15 DC19 DC22 DC23 DC27 | |
| HP03 | *bg* | | AII | | HC11 HC44 HC10 DC25 | |
| HP04 | *bg* | | C | | D01RBG D05RBG D33RBG D34RBG | |
| HP05 | *bg* | | D | | D28RBG D29RBG D30RBG D32RBG | |
| HP06 | *bg* | | AII | | HC12 AT27BG AT39BG | |
| HP07 | *bg* | | AII | | DC01 DC08 DC28 | |
| HP08 | *bg* | | D | | D02RBG D03RBG 07JBG | |
| HP09 | *bg* | | BIV | | HC46 HC47 | |
| HP10 | *bg* | | A | | AT09BG AT10BG | |
| HP11 | *bg* | | BIII | | HC07 | |
| HP12 | *bg* | | A | | HC27 | |
| HP13 | *bg* | | AII | | HC29 | |
| HP14 | *bg* | | BIV | | HC32 | |
| HP15 | *bg* | | BIII | | HC33 | |
| HP16 | *bg* | | BIV | | HC39 | |
| HP17 | *bg* | | BIV | | HC45 | |
| HP18 | *bg* | | AII | | HC49 | |
| HP19 | *bg* | | AII | | HC51 | |
| HP20 | *bg* | | AII | | HC09 | |
| HP21 | *bg* | | B | | HC34 | |
| HP22 | *bg* | | A | | DC26 | |
| HP23 | *bg* | | AII | | DC20 | |
| HP24 | *bg* | | D | | D04RBG | |
| HP25 | *bg* | | D | | D06RBG | |
| HP26 | *bg* | | D | | D31RBG | |
| HP27 | *bg* | | D | | D35RBG | |
| HP28 | *bg* | | C | | D36RBG | |
| HP29 | *bg* | | D | | VET01BG | |
| HP30 | *bg* | | E | | VET06BG | |
| HP31 | *bg* | | D | | AT01BG | |
| HP32 | *bg* | | AII | | AT11BG | |
| HP33 | *bg* | | AII | | AT14BG | |
| HP34 | *bg* | | AII | | AT21BG | |
| HP35 | *bg* | | AII | | 14JBG | |
| HP36 | *bg* | | AII | | 29JBG | |

**Table S4.** Access numbers of the reference sequences for *G. duodenalis* genetic assemblages obtained from GenBank

| **Genetic Assemblage** | ***gdh*** | ***bg*** | ***tpi*** |
| --- | --- | --- | --- |
| AI | L40509 | M36728.1 | L02120.1 |
| AII | L40510 | AY072723.1 | U57897.1 |
| AIII | - | DQ648777.1 | DQ650648.1 |
| BIII | AF069059.1 | AY072726.1 | AY228628.1 |
| BIV | L40508.1 | AY072725.1 | L02116.1 |
| C | U60985.1 | JF422719.1 | AY228641.1 |
| D | U60986.2 | AY545647.1 | DQ246216.1 |
| E | AY178740 | AY072729.1 | AY655705.1 |
| F | AY178744 | AY647264.1 | AF069558.1 |
| G | AY178748.1 | EU769221.1 | EU781013.1 |
| *G. muris* | - | EF455599.1 | AF069565.1 |
| *G. ardeae* | AF069060.2 | - | AF069564.1 |
| *G. microti* | - | - | AY228649.1 |

**Table S5.** Molecular characterization and accession numbers of clinical sequences obtained from hospital based on sequencing data from *gdh*, *bg*, and *tpi* genes.

| **Sample** | ***gdh*** | ***GenBank*** | ***bg*** | ***GenBank*** | ***tpi*** | ***GenBank*** |
| --- | --- | --- | --- | --- | --- | --- |
| HC01 | A | JN116442 | - | - | AII | KF922892 |
| HC02 | A | JN116443 | - | - | C | KF922893 |
| HC04 | - | - | - | - | C | KF922894 |
| HC06 | - | - | - | - | AII | KF922895 |
| HC07 | B | JN116444 | BIII | KF922976 | BIV | KF922896 |
| HC08 | - | - | - | - | C | KF922897 |
| HC09 | - | - | AII | KF922977 | BIV | KF922898 |
| HC10 | A | JN116445 | AII | KF922978 | AII | KF922899 |
| HC11 | A | KF923021 | AII | KF922979 | AII | KF922900 |
| HC12 | A | JN116446 | AII | KF922980 | AII | KF922901 |
| HC13 | A | JN116447 | - | - | C | KF922902 |
| HC14 | - | - | - | - | BIV | KF922903 |
| HC15 | B | JN116448 | - | - | BIV | KF922904 |
| HC16 | B | JN116449 | - | - | BIV | KF922905 |
| HC17 | - | - | - | - | BIV | KF922906 |
| HC18 | A | JN116450 | - | - | - | - |
| HC19 | - | - | - | - | C | KF922907 |
| HC20 | - | - | - | - | BIV | KF922908 |
| HC21 | B | JN116451 | - | - | AII | KF922909 |
| HC22 | A | JN116452 | - | - | AII | KF922910 |
| HC23 | B | JN116453 | - | - | AII | KF922911 |
| HC24 | - | - | - | - | BIV | KF922912 |
| HC25 | B | JN116454 | B | KF922981 | BIV | KF922913 |
| HC27 | A | JN116455 | A | KF922982 | AII | KF922914 |
| HC28 | A | JN116456 | - | - | - | - |
| HC29 | - | - | AII | KF922983 | AII | KF922915 |
| HC30 | B | JN116457 | - | - | BIV | KF922916 |
| HC31 | A | JN116458 | AII | KF922984 | AII | KF922917 |
| HC32 | B | JN116459 | BIV | KF922985 | BIV | KF922918 |
| HC33 | B | JN116460 | BIII | KF922986 | BIII | KF922919 |
| HC34 | B | JN116461 | B | KF922987 | BIV | KF922920 |
| HC35 | A | JN116462 | - | - | AII | KF922921 |
| HC36 | A | JN116463 | AII | KF922988 | AII | KF922922 |
| HC37 | - | - | - | - | BIV | KF922923 |
| HC38 | B | JN116464 | - | - | BIV | KF922924 |
| HC39 | B | JN116465 | BIV | KF922989 | BIV | KF922925 |
| HC40 | A | JN116466 | AII | KF922990 | AII | KF922926 |
| HC41 | B | JN116467 | - | - | BIV | KF922927 |
| HC42 | A | JN116468 | AII | KF922991 | AII | KF922928 |
| HC43 | B | JN116469 | - | - | BIV | KF922929 |
| HC44 | A | JN116470 | AII | KF922992 | AII | KF922930 |
| HC45 | B | JN116471 | BIV | KF922993 | BIV | KF922931 |
| HC46 | B | JN116472 | BIV | KF922994 | BIV | KF922932 |
| HC47 | B | JN116473 | BIV | KF922995 | BIV | KF922933 |
| HC48 | A | JN116474 | AII | KF922996 | AII | KF922934 |
| HC49 | A | JN116475 | AII | KF922997 | AII | KF922935 |
| HC50 | A | JN116476 | AII | KF922998 | AII | KF922936 |
| HC51 | A | JN116477 | AII | KF922999 | AII | KF922937 |
| HC43A* | - | - | - | - | BIV | KM495706 |
| HC43B* | - | - | - | - | BIV | KM495707 |
| HC43C* | - | - | - | - | BIV | KM495708 |

*Sequences derived from molecular cloning of HC43 isolate

**Table S6.** Molecular characterization and accession numbers of sequences obtained from day-care center based on sequencing data from *gdh*, *bg*, and *tpi* genes.

| **Sample** | ***gdh*** | ***GenBank*** | ***bg*** | ***GenBank*** | ***tpi*** | ***GenBank*** |
| --- | --- | --- | --- | --- | --- | --- |
| DC01 | B | JN116478 | AII | KF923000 | BIV | KF922938 |
| DC02 | - | - | AII | KF923001 | BIV | KF922939 |
| DC03 | B | JN116479 | AII | KF923002 | BIV | KF922940 |
| DC04 | B | JN116480 | - | - | BIII | KF922941 |
| DC05 | B | JN116481 | AII | KF923003 | BIV | KF922942 |
| DC06 | - | - | - | - | BIV | KF922943 |
| DC07 | A | JN116482 | AII | KF923004 | - | - |
| DC08 | - | - | AII | KF923005 | BIV | KF922944 |
| DC09 | B | JN116483 | - | - | BIII | KF922945 |
| DC10 | - | - | - | - | BIII | KF922946 |
| DC11 | - | - | AII | KF923006 | BIII | KF922947 |
| DC12 | A | JN116484 | AII | KF923007 | AII | KF922948 |
| DC13 | A | JN116485 | AII | KF923008 | - | - |
| DC14 | - | - | AII | KF923009 | BIV | KF922949 |
| DC15 | A | JN116486 | AII | KF923010 | AII | KF922950 |
| DC16 | B | JN116487 | - | - | BIV | KF922951 |
| DC17 | B | JN116488 | - | - | BIV | KF922952 |
| DC18 | - | - | - | - | BIII | KF922953 |
| DC19 | B | JN116489 | AII | KF923011 | BIV | KF922954 |
| DC20 | B | JN116490 | AII | KF923012 | BIV | KF922955 |
| DC21 | B | JN116491 | - | - | BIV | KF922956 |
| DC22 | B | JN116492 | AII | KF923013 | BIV | KF922957 |
| DC23 | - | - | AII | KF923014 | BIII | KF922958 |
| DC24 | - | - | - | - | BIV | KF922959 |
| DC25 | A | JN116493 | AII | KF923015 | AII | KF922960 |
| DC26 | - | - | A | KF923016 | - | - |
| DC27 | A | JN116494 | AII | KF923017 | AII | KF922961 |
| DC28 | A | JN116495 | AII | KF923018 | AII | KF922962 |

**Table S7.** Molecular characterization and accession numbers of sequences obtained from environmental isolates based on sequencing data from *gdh* and *tpi* genes.

| **Sample** | **Source** | ***gdh*** | ***GenBank*** | ***tpi*** | ***GenBank*** |
| --- | --- | --- | --- | --- | --- |
| ENV01 | SWWTP | A | JN116502 | - | - |
| ENV02 | Water Abstraction | - | - | C | KF922968 |
| ENV03 | Proença Stream | - | - | BIV | KF922969 |
| ENV04 | Serafim Stream | - | - | BIII | KF922970 |
| ENV05 | Anhumas River | D | JN116503 | BIV | KF922971 |
| ENV06 | Hospital Sewage | B | JN116504 | BIII | KF922972 |

**Table S8.** Molecular characterization and accession numbers of sequences obtained from sewage based on sequencing data from *tpi* and *bg* genes.

| **Isolate** | **Source** | ***tpi*** | ***GenBank*** | ***bg*** | ***GenBank*** |
| --- | --- | --- | --- | --- | --- |
| 1J | Hospital | B | KT728542 | - | - |
| 2J | Hospital | A | KT728543 | - | - |
| 3J | Hospital | C | KT728544 | - | - |
| 4J | Hospital | AII | KT728545 | - | - |
| 7J | Hospital | AII | KT728546 | D | KT728528 |
| 8J | Hospital | BIV | KT728547 | - | - |
| 10J | WWTP | C | KT728548 | - | - |
| 12J | WWTP | AII | KT728549 | - | - |
| 13J | WWTP | AII | KT728550 | - | - |
| 14J | WWTP | AII | KT728551 | AII | KT728529 |
| 15J | WWTP | AII | KT728552 | AII | KT728530 |
| 16J | WWTP | C | KT728553 | AII | KT728531 |
| 17J | WWTP | AII | KT728554 | - | - |
| 18J | WWTP | AII | KT728555 | - | - |
| 19J | WWTP | AII | KT728556 | - | - |
| 20J | WWTP | C | KT728557 | - | - |
| 21J | WWTP | AII | KT728558 | - | - |
| 22J | Hospital | B | KT728559 | - | - |
| 23J | Hospital | AII | KT728560 | - | - |
| 25J | WWTP | AII | KT728561 | - | - |
| 26J | WWTP | AII | KT728562 | - | - |
| 27J | WWTP | AII | KT728563 | - | - |
| 28J | WWTP | BIV | KT728564 | - | - |
| 29J | WWTP | BIV | KT728565 | AII | KT728532 |
| 30J | WWTP | AII | KT728566 | - | - |
| 31J | Hospital | AII | KT728567 | - | - |
| 33J | Hospital | AII | KT728568 | AII | KT728533 |
| 35J | Hospital | AII | KT728569 | - | - |
| 36J | WWTP | C | KT728570 | - | - |
| 37J | WWTP | AII | KT728571 | - | - |

WWTP sequences came from WWTP Piçarrão in the city of Campinas. Hospital sequences came from the sewage from the Hospital of the University of Campinas (UNICAMP)

**Table S9.** Molecular characterization and accession numbers of sequences obtained from Atibaia River based on sequencing data from *bg* gene.

| **Isolate** | **Source** | ***bg*** | ***GenBank*** |
| --- | --- | --- | --- |
| AT01 | Atibaia River | D | KT728418 |
| AT02 | Atibaia River | A | KT728419 |
| AT03 | Atibaia River | A | KT728420 |
| AT04 | Atibaia River | AII | KT728421 |
| AT06 | Atibaia River | AII | KT728422 |
| AT07 | Atibaia River | AII | KT728423 |
| AT08 | Atibaia River | A | KT728424 |
| AT09 | Atibaia River | A | KT728425 |
| AT10 | Atibaia River | A | KT728426 |
| AT11 | Atibaia River | AII | KT728427 |
| AT12 | Atibaia River | A | KT728428 |
| AT13 | Atibaia River | AII | KT728429 |
| AT14 | Atibaia River | AII | KT728430 |
| AT15 | Atibaia River | A | KT728431 |
| AT16 | Atibaia River | AII | KT728432 |
| AT17 | Atibaia River | AII | KT728433 |
| AT19 | Atibaia River | AII | KT728434 |
| AT20 | Atibaia River | AII | KT728435 |
| AT21 | Atibaia River | AII | KT728436 |
| AT22 | Atibaia River | AII | KT728437 |
| AT24 | Atibaia River | A | KT728438 |
| AT25 | Atibaia River | AII | KT728439 |
| AT26 | Atibaia River | AII | KT728440 |
| AT27 | Atibaia River | A | KT728441 |
| AT29 | Atibaia River | A | KT728442 |
| AT30 | Atibaia River | AII | KT728443 |
| AT31 | Atibaia River | A | KT728444 |
| AT32 | Atibaia River | A | KT728445 |
| AT33 | Atibaia River | AII | KT728446 |
| AT34 | Atibaia River | A | KT728447 |
| AT35 | Atibaia River | A | KT728448 |
| AT37 | Atibaia River | AII | KT728449 |
| AT38 | Atibaia River | AII | KT728450 |
| AT39 | Atibaia River | A | KT728451 |
| AT40 | Atibaia River | AII | KT728452 |
| AT41 | Atibaia River | A | KT728453 |
| AT42 | Atibaia River | AII | KT728454 |
| AT43 | Atibaia River | AII | KT728455 |
| AT44 | Atibaia River | AII | KT728456 |
| AT45 | Atibaia River | AII | KT728457 |

**Table S10.** Molecular characterization and accession numbers of sequences obtained from veterinary isolates based on sequencing data from *tpi* gene.

| **Isolate** | **Source** | ***tpi*** | **GenBank** | ***tpi L*** | **GenBank** |
| --- | --- | --- | --- | --- | --- |
| D1 | Dog | C | KT728505 | C | KT728473 |
| D2 | Dog | - | - | D | KT728474 |
| D3 | Dog | C | KT728506 | D | KT728475 |
| D4 | Dog | C | KT728507 | D | KT728476 |
| D5 | Dog | C | KT728508 | D | KT728477 |
| D6 | Dog | C | KT728509 | D | KT728478 |
| D7 | Dog | C | KT728510 | D | KT728479 |
| D8 | Dog | C | KT728511 | D | KT728480 |
| D9 | Dog | C | KT728512 | D | KT728481 |
| D10 | Dog | C | KT728513 | D | KT728482 |
| D11 | Dog | C | KT728514 | C | KT728483 |
| D12 | Dog | - | - | D | KT728484 |
| D13 | Dog | - | - | D | KT728485 |
| D15 | Dog | - | - | D | KT728486 |
| D16 | Dog | C | KT728515 | C | KT728487 |
| D17 | Dog | C | KT728516 | - | - |
| D18 | Dog | C | KT728517 | D | KT728488 |
| D19 | Dog | C | KT728518 | C | KT728489 |
| D20 | Dog | C | KT728519 | - | - |
| D21 | Dog | C | KT728520 | D | KT728490 |
| D22 | Dog | - | - | D | KT728491 |
| D23 | Dog | - | - | D | KT728492 |
| D25 | Dog | - | - | D | KT728493 |
| D26 | Dog | - | - | D | KT728494 |
| D27 | Dog | C | KT728521 | D | KT728495 |
| D28 | Dog | - | - | D | KT728496 |
| D29 | Dog | C | KT728522 | D | KT728497 |
| D30 | Dog | - | - | D | KT728498 |
| D31 | Dog | - | - | D | KT728499 |
| D32 | Dog | - | - | D | KT728500 |
| D34 | Dog | C | KT728523 | - | - |
| D35 | Dog | C | KT728524 | D | KT728501 |
| D37 | Dog | C | KT728525 | - | - |
| D38 | Dog | - | - | D | KT728502 |
| D39 | Dog | - | - | D | KT728503 |
| D40 | Dog | - | - | D | KT728504 |
| D41 | Dog | C | KT728526 | C | - |
| VET01 | Dog | BIV | KF922963 | D | KF922973 |
| VET02 | Dog | AII | KF922964 | C | KF922974 |
| VET04 | Cat | AII | KF922965 | - | - |
| VET05 | Cat | BIV | KF922966 | D | KF922975 |
| VET06 | Calf | AII | KF922967 | - | - |
| VET02LA* | Dog | - | - | D | KF922973 |
| VET02LB* | Dog | - | - | C | KF922974 |
| VET02LC* | Dog | - | - | D | KF922975 |

*tpi L* refers to primers developed by Lebbad *et al, 2010 Vet Parasitol 168: 231–239.*

**Table S11.** Molecular characterization and accession numbers of sequences obtained from veterinary isolates based on sequencing data from *bg* and *gdh* genes.

| **Isolate** | **Source** | ***bg*** | **GenBank** | ***gdh*** | **GenBank** |
| --- | --- | --- | --- | --- | --- |
| D1 | Dog | C | KT728458 | C | KT728534 |
| D2 | Dog | D | KT728459 | D | KT728535 |
| D3 | Dog | D | KT728460 | C | KT728536 |
| D4 | Dog | D | KT728461 | D | KT728537 |
| D5 | Dog | C | KT728462 | C | KT728538 |
| D6 | Dog | D | KT728463 | D | KT728539 |
| D7 | Dog | - | - | D | KT728540 |
| D8 | Dog | - | - | D | KT728541 |
| D28 | Dog | D | KT728464 | - | - |
| D29 | Dog | D | KT728465 | - | - |
| D30 | Dog | D | KT728466 | - | - |
| D31 | Dog | D | KT728467 | - | - |
| D32 | Dog | D | KT728468 | - | - |
| D33 | Dog | C | KT728469 | - | - |
| D34 | Dog | C | KT728470 | - | - |
| D35 | Dog | D | KT728471 | - | - |
| D36 | Dog | C | KT728472 | - | - |
| VET01 | Dog | D | KF923019 | D | JN116498 |
| VET02 | Dog | - | - | D | JN116499 |
| VET03 | Dog | - | - | C | JN116500 |
| VET05 | Cat | - | - | D | JN116497 |
| VET06 | Calf | E | KF923020 | E | JN116496 |
